# *Trichuris trichiura*—An Unwelcome Surprise during Colonoscopy

**DOI:** 10.4269/ajtmh.18-0209

**Published:** 2018-09

**Authors:** Tagore Sunkara, Santosh R. Sharma, Andrew Ofosu

**Affiliations:** Division of Gastroenterology and Hepatology, The Brooklyn Hospital Center, Brooklyn, New York

**Figure 1.**
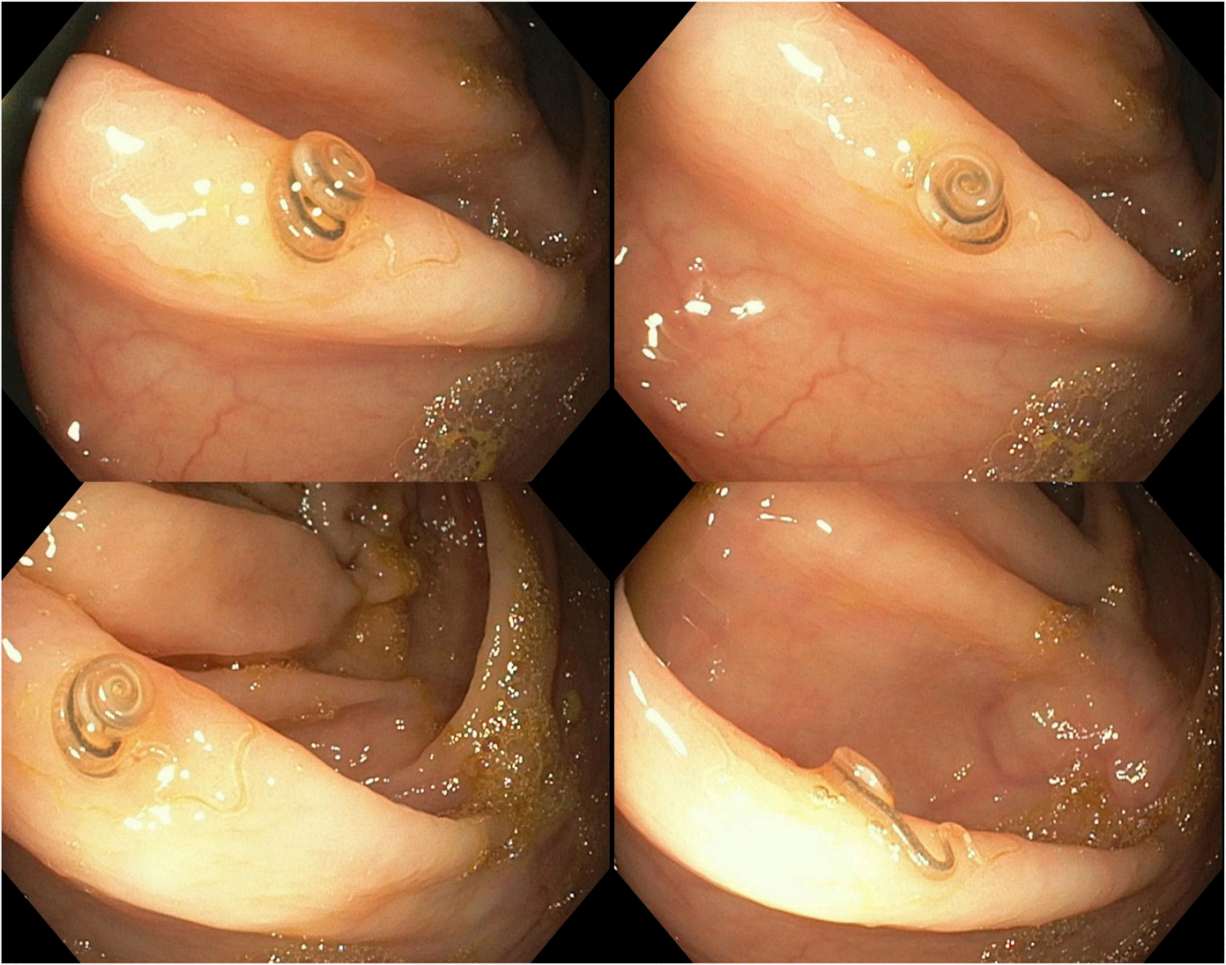
Colonoscopy revealed a brown-colored live worm on the ileocecal valve. This figure appears in color at www.ajtmh.org.

A 50-year-old female who immigrated from Bangladesh about 15 years ago was referred to the gastroenterology clinic for 20 pounds weight loss over a period of 2 months. The physical examination was normal and laboratory tests were significant for mild eosinophilia. As a part of weight loss workup, colonoscopy was performed and revealed a brown-colored live worm on the ileocecal valve (Figure 1). The worm was retrieved with cold biopsy forceps and sent to pathology for identification. On microscopy, it was identified as *Trichuris trichiura*. The patient was treated with albendazole 400 mg for 3 days. Family members were recommended to be tested for stool ova and parasites.

Helminthic infestations are common in preschool and school-going children in tropical and subtropical countries; however, cases have been seen in non-endemic areas mostly because of migration.^1^ *Trichuris trichiura*, also known as whipworm, is the most commonly diagnosed helminth during colonoscopy.^2^ Mode of transmission to humans is by ingesting eggs of the helminths. After ingestion, the eggs are hatched into larvae in the small intestine, which subsequently grow into mature forms and localize in the colon.^3^ Most patients infested with whipworm have mild to no symptoms. Symptoms if present are chronic abdominal pain, anorexia, diarrhea, and weight loss.^2^ Serious side effects such as colonic obstruction and lower gastrointestinal bleeding have been reported with increased parasite load.^4^ Heavy infestation with the worms can lead to malnutrition, rectal prolapse, and seldom “whipworm dysentery syndrome” manifested as diarrhea, anemia, and malnutrition.^3^ Diagnosis is usually made by stool examination, which demonstrates the characteristic barrel-shaped ova in the stool.^3^ Antihelminthic agents are the mainstay of treatment; however, removal with forceps during colonoscopy may be needed if the worms are embedded within the mucosa and difficult to expel.^5^
